# Unveiling the Antioxidant Mechanism of Canolol: Packaging Impacts the Long-Term Stability of Microwave-Pretreated Rapeseed Oil

**DOI:** 10.3390/foods15101797

**Published:** 2026-05-19

**Authors:** Ying Huang, Chang Zheng, Changsheng Liu, Chuyun Wan, Qianchun Deng, Zisong Li, Mingming Zheng

**Affiliations:** 1 Oil Crops Research Institute, Chinese Academy of Agricultural Sciences, Key Laboratory of Oilseeds Processing, Ministry of Agriculture and Rural Affairs of the People’s Republic of China, Oil Crops and Lipids Process Technology National & Local Joint Engineering Laboratory, Hubei Key Laboratory of Lipid Chemistry and Nutrition, Wuhan 430062, China; huangying01@caas.cn (Y.H.); zhengchang@caas.cn (C.Z.); liuchangsheng@caas.cn (C.L.); wanchuyun@caas.cn (C.W.); dengqianchun@caas.cn (Q.D.); 2 National Nanfan Research Institute (Sanya), Chinese Academy of Agricultural Sciences, Sanya 572024, China; 3 Shandong Xingquan Oil Co., Ltd., Linyi 276600, China; 13953905179@163.com

**Keywords:** rapeseed, microwave pretreatment, canolol, antioxidant mechanism, packaging strategy, storage stability

## Abstract

Canolol is a pivotal phenolic antioxidant in rapeseed oil, yet its specific antioxidant mechanism and stability determinants during storage remain poorly understood. This study elucidates the antioxidant pathway of canolol within a lipid autoxidation model and evaluates its stability during the 52-week storage (25 ± 2 °C) of microwave-pretreated rapeseeds under varying packaging conditions. Rapeseeds were packaged in polyamide/polyethylene (PA/PE) vacuum bags and polypropylene (PP) atmospheric bags, and then monitored for seed quality, oil oxidative indices, and micronutrient contents. Via high-performance liquid chromatography-quadrupole time-of-flight tandem mass spectrometry (HPLC-Q-TOF/MS/MS), a canolol-derived dimeric oxidation product (C_20_H_24_O_7_, *m*/*z* 375.1437) was tentatively identified in an 2,2’-azobis(isobutyronitrile) (AIBN)-initiated ethyl linoleate (EtL) autoxidation system. The MS/MS fragmentation pattern—characterized by neutral H_2_O loss, sequential •CH_3_ eliminations, and syringyl-type diagnostic ions—supports a mechanism involving hydrogen atom transfer (HAT) from canolol to lipid-derived peroxyl radicals. This is followed by the oxidative cross-coupling of a canolol-derived phenoxyl radical (ArO•) with a hydroxyethylated intermediate (Ar′O•), confirming canolol’s role as a chain-breaking antioxidant. Correlation analyses confirmed canolol as the primary antioxidant (*r* = −0.914, −0.984/−0.959, −0.883 with acid value/peroxide value, *p* < 0.01), with a synergistic effect relationship with tocopherols (*r* = 0.878, 0.966, *p* < 0.01). PA/PE vacuum packaging (low oxygen permeability) significantly mitigated canolol degradation (22.41% loss vs. 76.34% in PP), reducing tocopherol loss and oil oxidation. This study clarifies canolol’s antioxidant pathway in rapeseed oil, providing theoretical insights for phenolic antioxidant research and practical packaging guidance for the edible oil industry.

## 1. Introduction

Rapeseed (*Brassica napus* L.) represents one of the most economically important oilseed crops globally, with extensive cultivation across China, Canada, India, and Europe. Among common edible oils, rapeseed oil is distinguished by its favorable nutritional profile, characterized by a low saturated fatty acid content (~6%) and optimal n-6/n-3 polyunsaturated fatty acid (PUFA) ratio (~2:1), considered beneficial for human cardiovascular health. Furthermore, unrefined rapeseed oil serves as a rich source of bioactive constituents, including phytosterols (4840–7620 mg/kg) and tocopherols (486–913 mg/kg) [1]. Phenolic compounds constitute a major class of phytochemicals with significant biological activity, particularly in quenching radical reactions responsible for lipid oxidation [2], among which sinapic acid and its derivatives have emerged as key antioxidants in rapeseed. Notably, sinapic acid extracts isolated from rapeseed by-products (500 μmol/kg) have demonstrated superior efficacy in inhibiting lipid oxidation in bulk oil systems compared to mixed tocopherols (*α*/*γ* ratio of 3.5:1, 500 μmol/kg) [3]. Under high-temperature processing conditions such as microwave pretreatment, sinapic acid undergoes decarboxylation to form canolol (2,6-dimethoxy-4-vinylphenol) [4]. This compound is considered responsible for the enhanced stability of crude rapeseed oils, contributing to their improved thermal stability during frying [5,6] and a marked reduction in lipid oxidation rates under accelerated storage conditions [7,8].

Proper rapeseed storage is paramount for preventing oil oxidation and maintaining oil quality, thereby ensuring a stable supply of edible oil with storage efficacy governed by packaging type, storage conditions, and raw material quality. Vacuum packaging has gained prominence as an effective preservation strategy, primarily by inhibiting mold growth, pest development, and respiratory metabolism through limiting oxygen access, with the superiority of multi-layer vacuum packaging materials demonstrated in the literature. Chun et al. [9] demonstrated that vacuum storage reduced *α*-tocopherol loss in dry-roasted peanuts to approximately 50% after 12 weeks, compared with ~90% loss under atmospheric conditions, while Severini et al. [10] emphasized that effective vacuum conditions provided by high-oxygen-barrier packaging films are essential for preserving roasted almond quality, as evidenced by peroxide value (POV), triglyceride oligopolymer content, and oxidized triglyceride assessments. Fu et al. [11] further reported that polyethylene terephthalate (PET)/aluminum (AL)/polyamide (PA)/polyethylene (PE) and PA/PE bags significantly outperformed conventional PE and woven bags in maintaining peanut freshness, preventing insect infestation, and controlling aflatoxin contamination. Rapeseed storage efficacy can be further improved via targeted pre-storage interventions. Specifically, microwave pretreatment prior to oil extraction significantly increases bioactive phytochemical recovery [12,13,14] while promoting the formation of beneficial compounds such as canolol and Maillard reaction products [7]. These compositional changes improve oxidative stability, positioning microwave pretreatment as a viable strategy to prolong commercial rapeseed oil’s shelf life.

Furthermore, the critical roles of endogenous antioxidants in delaying lipid oxidation have been well-documented. Zheng et al. [8] established significant linear correlations between canolol content and oxidative indices, including POV and *p*-anisidine value (*p*-AV), highlighting canolol as a critical determinant of lipid oxidation progression. Similarly, Abramovič et al. [15] reported that POV increased linearly with decreasing phenolic content, with correlation coefficients as high as *r* = −0.996 and −0.997, underscoring phenolic compounds’ antioxidant efficacy. In addition, Pan et al. [16] demonstrated strong negative correlations between *α*-/*γ*-tocopherol, total tocopherols, and oxidation products in vegetable oils. Consequently, concurrently monitoring these micronutrients alongside classical oxidation indices is essential to comprehensively evaluate the storage stability of vegetable oils. Despite these advances, significant knowledge gaps remain. The regulatory effect of different packaging materials—specifically vacuum PA/PE vs. atmospheric polypropylene (PP)—on canolol stability during long-term storage is currently unexplored. More importantly, while the radical-scavenging capacity of canolol is established, the specific oxidation products formed when canolol intercepts peroxyl radicals, and the precise molecular pathway of its protective mechanism, have not been characterized. This mechanistic ambiguity limits the rational optimization of processing and storage protocols to maximize oil stability. Beyond canolol, the stability of tocopherols and phytosterols during storage is subject to oxidative stress [17,18], potentially linked to canolol’s antioxidant behavior.

Aiming to address the above-mentioned research gaps, we focus on canolol’s antioxidant activity and oxidative degradation mechanism, specifically targeting the following objectives: (1) to compare the storage stability of microwave-pretreated rapeseed and its cold-pressed oil in PA/PE vacuum vs. PP atmospheric packing over 52 weeks; (2) to monitor the degradation evolutions of key micronutrients (canolol, tocopherols, phytosterols) and elucidate the correlation between canolol stability and classical oxidative indices (acid value, AV and POV); and (3) to simulate lipid peroxidation using an 2,2’-azobis(isobutyronitrile) (AIBN)-initiated ethyl linoleate (EtL) oxidation system and deduce canolol’s antioxidant mechanism by identifying its oxidation products via high-performance liquid chromatography-quadrupole time-of-flight tandem mass spectrometry (HPLC-Q-TOF/MS/MS). In this research, we seek to clarify the oxidative degradation pathway of canolol, enriching the theoretical framework for phenolic antioxidants in vegetable oils. Furthermore, the validation of packaging strategies for preserving canolol activity provides practical technical guidance for the long-term storage of rapeseed oil, holding substantial academic and industrial significance.

## 2. Materials and Methods

### 2.1. Materials

The rapeseed (*Brassica napus* L. cv. Zhongyouza19) was provided by the Oil Crop Research Institute, Chinese Academy of Agricultural Sciences (Wuhan, China), containing 5.88% moisture, 21.51% crude protein, 49.12% crude oil, 18.69% carbohydrate and 4.80% ash. Seeds were stored at 10 °C and 50% relative humidity prior to experiments.

The following compounds were procured from Sigma-Aldrich Co. (St. Louis, MO, USA): tocopherol standards (*α*- and *γ*-isomers), phytosterols (brassicasterol, campesterol and *β*-sitosterol), 5*α*-cholestane, N,O-Bis(trimethylsilyl)trifluoroacetamide with trimethylchlorosilane (BSTFA + TMCS), and Folin–Ciocalteu reagent (FC, 2 N), as well as 2,2-dipheny1-1-picrylhydrazyl (DPPH), 2,4,6-tris(2-pyridyl)-s-triazine (TPTZ), iron(III) chloride hexahydrate, and 6-hydroxy-2,5,7,8-tetramethylchroman-2-carboxylic acid (Trolox). Canolol standard was purchased from Apin Chemicals Limited (Abingdon, Oxon, UK); chromatographic-grade methanol, isopropanol, *n*-hexane, formic acid, acetic acid and acetonitrile were purchased from Merck (Darmstadt, Germany). AIBN and EtL were purchased from Aladdin Biochemical Technology Co., Ltd. (Shanghai, China). Other chemicals used in the study were of analytical grade and purchased from Sinopharm Chemical Regent Co., Ltd. (Shanghai, China).

### 2.2. Sampling and Storage Treatment

Rapeseed samples were subjected to microwave treatment using a WB6E microwave experimental apparatus (Nanjing Kaile Electric Microwave Equipment Co., Ltd., Nanjing, China) at 6 kW for 7 min, with the material thickness controlled at 1 cm and the treatment temperature maintained at 140 ± 5 °C. All tests were performed in triplicate to ensure data reliability. After natural cooling, the remaining microwaved rapeseeds (300 g) were packaged in either PA/PE multi-layer co-extrusion vacuum bags (L × W × THK = 25 × 20 × 0.19 cm) heat-sealed at 150 °C using a vacuum packaging machine (DZQ400/2SA, Wenzhou Zhonghuan Machinery Equipment Co., Ltd. Wenzhou, China), or PP bags (L × W × THK = 24 × 20 × 0.5 cm) at atmospheric pressure. During the one-year storage period at 25 ± 2 °C, three randomly selected subsamples were collected from each storage condition at weeks 1, 5, 11, 24, 40, and 52. The corresponding seed oils were pressed using a screw press (CA59G, German Monforts Group, Moenchen-gladbach, North Rhine-Westphalia, Germany) operated at a screw speed of 10 rpm with a power consumption of 0.2 kW and a nozzle diameter of 4 mm, while the temperature at the outlet of the press chamber was continuously maintained at 60 ± 2 °C, as monitored by a non-contact infrared thermometer. Subsequently, the collected oils were centrifuged at 25,230× *g* for 15 min (Avanti J-26 XP, Beckman Coulter Inc., Brea, CA, USA) under refrigeration (4 ± 2 °C), transferred to amber vials, and analyzed within three days. The initial AV and POV of the cold-pressed oil from raw seeds without microwave pretreatment and storage were determined as 1.16 mg KOH/g and 0.57 mmol O_2_/kg, respectively.

### 2.3. Physicochemical Parameters

Rapeseed and rapeseed oil’s physicochemical properties were analyzed using the indicated standard methods. For rapeseeds, moisture content was determined by oven drying at 105 °C to a constant weight according to the National Standard of China (GB 5009.3-2016) [19], and oil content was determined via Soxhlet extraction (gravimetric analysis) in accordance with GB 5009.6-2016 [20]. For rapeseed oil, AV was determined using the titration method described in AOCS Official Method cd 3d-63 [21], and POV was determined using the indicator titration method specified in GB 5009.227-2016 [22].

### 2.4. Determination of Total Phenolic Content (TPC) and Antioxidant Properties

An aliquot (0.5 g) of seed powder was mixed with 70% aqueous methanol (3 × 5 mL) using a vortex oscillator and centrifuge with the collected extraction stored in a sealed test tube at 4 °C in the dark; this was performed in triplicate. Diluted (1:2) extracting solution was added, alongside 0.5 mL of FC reagent and 1 mL of saturated sodium carbonate solution, and the volume was subsequently adjusted to 10 mL with pure water. After 60 min of dark reaction, the absorbance was measured at 765 nm with a DU 800 UV/visible spectrophotometer (Beckman Coulter, Brea, CA, USA), with sinapic acid used for calibration. The results were expressed as milligrams of sinapic acid equivalents per 100 g sample (mg SAE/100 g).

The DPPH radical scavenging activity of the rapeseed phenolic extract was evaluated following the procedure outlined by Wang et al. [23], with minor modifications. Specifically, 0.5 mL of the extract was mixed with 2.5 mL of a 9.64 × 10^−2^ M methanolic solution of DPPH radical. A control sample, in which the extract was replaced with an equal volume of methanol, was used to determine the initial DPPH absorbance. After incubation in the dark for 30 min, the absorbance was measured at 515 nm to quantify the remaining DPPH radical. The antioxidant capacity was expressed as micromoles of Trolox equivalents per 100 g of sample (μmol TE/100 g).

The ferric reducing antioxidant power (FRAP) assay of the rapeseed phenolic extract was conducted according to Wang et al.’s method [23]. The FRAP working solution was prepared by mixing 2.5 mL of 3.1 mg/mL TPTZ in HCl, 2.5 mL of 20 mM FeCl_3_, and 25 mL of 0.1 M acetate buffer (pH 3.6), followed by incubation in a 37 °C water bath for 10 min. Subsequently, 0.5 mL of the extract was combined with 2 mL of the working solution and diluted to a final volume of 10 mL with distilled water. This mixture was vortexed vigorously for 30 s and incubated in the dark for 20 min, after which absorbance was measured at 593 nm. A blank sample containing 0.5 mL of methanol in place of the extract was prepared and processed under the same conditions. The results were expressed as micromoles of Trolox equivalent per 100 g of sample (μmol TE/100 g).

### 2.5. Characterization of Lipid Micronutrients

#### 2.5.1. Tocopherol Measurement

Tocopherols were analyzed following the high-performance liquid chromatography (HPLC) method described by Ma et al. [24] with minor adaptations. Briefly, 2.0 g of oil was accurately weighed, dissolved in *n*-hexane, and transferred to a 25-mL brown volumetric flask, which was then made up to volume with the same solvent. A 20 μL aliquot of the solution was then injected into an analytical silica column (250 mm × 4.6 mm, 5 μm) maintained at 30 °C. Separation was carried out isocratically using a mobile phase consisting of *n*-hexane and isopropanol (99.5:0.5, *v*/*v*) at a flow rate of 1.0 mL/min, and detection was performed with a diode-array detector (SPD-M20A, Shimadzu, Tokyo, Japan) set at 292 nm and 298 nm for tocopherols. By comparing the results with the chromatograms of *α*- and *γ*-tocopherol standards, each tocopherol in the analyzed oil samples was identified and quantified.

#### 2.5.2. Phytosterol Measurement

According to the method described by Wang et al. [25], unsaponifiable matter was prepared as followed. An oil sample (~20 mg) was weighed and spiked with 0.5 mL of 5*α*-cholestanol solution (0.5 mg/mL) as an internal standard, and the mixture was combined with 10 mL of 2 M KOH in 95% ethanol in a ground-glass tube and saponified in a water bath at 60 °C for 60 min with periodic shaking. After cooling, 4 mL of deionized water was added, followed by triple extraction with 10 mL of *n*-hexane each. After centrifugation at 5000 rpm for 5 min, the hexane layer containing the unsaponifiable fraction was collected and concentrated to approximately 1 mL under a gentle nitrogen stream. Derivatization was performed by adding 200 μL of BSTFA + TMCS (99:1, *v*/*v*) to the concentrated extract and heating at 105 °C for 15 min. Gas chromatography (GC) analysis was carried out on a DB-5HT capillary column (30 m × 0.22 mm × 0.1 μm; Agilent, Santa Clara, CA, USA) with helium as the carrier gas at a constant flow rate of 1.5 mL/min, an injector temperature of 280 °C with a split ratio of 25:1, and an injection volume of 1 μL. In the oven, an initial temperature of 60 °C (held for 1 min) was increased at 40 °C/min to 310 °C, and held for 10 min. Sterols were identified by comparing their retention times with those of authentic standards and quantified based on the internal standard method. Results were expressed in milligrams per kilogram of oil (mg/kg).

#### 2.5.3. Canolol Measurement

Following the procedure previously reported by Huang et al. [26], the oil sample’s phenolic extract was obtained by liquid–liquid extraction with 80% methanol. The extract was then filtered through a 0.45-μm membrane and immediately analyzed using an Acquity ultra-performance liquid chromatography system (UPLC, Waters, Milford, CT, USA) coupled with a photodiode array (PDA) detector. Chromatographic separation was performed on an Acquity UPLC BEH C18 column (2.1 × 100 mm, 1.7 μm; Waters, Milford, CT, USA), and the autosampler and column temperatures were maintained at 20 °C and 30 °C, respectively. The mobile phase consisted of methanol (solvent A) and 2% acetic acid in water (solvent B), delivered at a flow rate of 0.21 mL/min under the following gradient program: 0–7.4 min, 95–75% B; 7.4–10.07 min, 75–71% B; 10.07–16.73 min, 71–64% B; 16.73–23.4 min, 64–55% B; 23.4–25.4 min, 55–35% B; 25.4–27.4 min, 35–95% B; 27.4–30.4 min, 95% B; 30.4–35 min, 95% B. The injection volume was 3 μL, and detection was carried out at 270 nm. Quantification was based on an external calibration curve of canolol, which showed good linearity (*R*^2^ = 0.9983) over the concentration range of 1–500 μg/mL, and results were expressed as milligrams of canolol per kilogram of oil (mg/kg).

### 2.6. HPLC-Q-TOF/MS/MS Detection of Oxidation Products from Canolol and EtL Hydroperoxides in EtL Oxidation System

The identification of canolol and its oxidation products was performed using a liquid chromatography system (LC-20, Shimadzu, Japan) coupled with a hybrid quadrupole time-of-flight mass spectrometer (Triple TOF 5600+, AB Sciex, Framingham, MA, USA). Chromatographic separation was achieved on a Polar C18 column (Luna Omega, 100 mm × 2.1 mm, 3 µm; Phenomenex, Torrance, CA, USA) maintained at 40 °C with a mobile phase comprising 0.1% formic acid in water (A) and acetonitrile (B) at a flow rate of 0.21 mL/min and injection volume of 3 µL. A gradient elution program was employed as follows: 0–0.01 min, 5% B; 0.01–7.4 min, 5–25% B; 7.4–10.07 min, 25–29% B; 10.07–16.73 min, 29–36% B; 16.73–23.40 min, 36–45% B; 23.40–25.40 min, 45–65% B; 25.40–27.40 min, 65–5% B. This was followed by a re-equilibration step with 5% B from 27.40 to 30.40 min, and the procedure was maintained for 35 min.

Mass spectrometry was operated in both positive and negative electrospray ionization (ESI) modes across a mass range of 70 to 2000 *m*/*z*. The main parameters were as follows: ion gas 1 (GS1) and 2 (GS2) were held at 55 psi and 60 psi, respectively, the curtain gas was kept at 30 psi, and the ion temperature was 600 °C, ion spray voltage floating was 5500 V/−4500 V, collision energy was ±35 V, collision energy spread was ±15 V, and declustering potential was 100 V. Nitrogen was used as the nebulizer and auxiliary gas, and target compound collection was conducted via LC-PDA at 270 nm.

### 2.7. Statistical Analysis

The results are presented as means ± standard deviation from three replicates of each experiment. Statistical analyses and data visualization were conducted using Excel 2016 and Origin 8.5, respectively, and statistical significance (*p* < 0.05) was assessed using SPSS 24.0.

## 3. Results and Discussion

### 3.1. The Moisture and Oil Content of Microwaved Rapeseeds in PP and PA/PE Packaging During Storage

**Figure 1:**
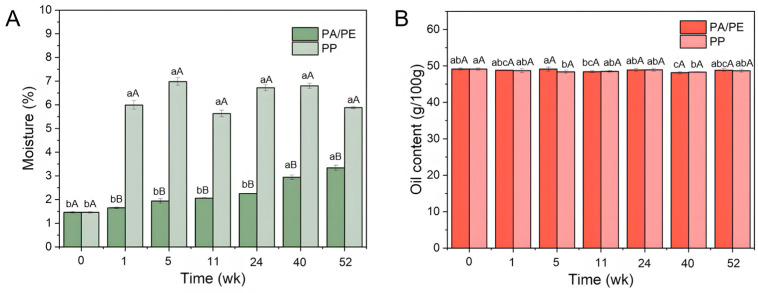
The moisture (**A**) and oil content (**B**) of rapeseeds during 13-month storage in PA/PE and PP packaging. Note: Different lowercase letters indicate significant (*p* < 0.05) differences over different storage periods among samples stored in the same packaging materials, while different uppercase letters indicate significant differences between different packaging materials during the same storage period (*p* < 0.05).

To evaluate the protective efficacy of different packaging materials, we monitored the changes in rapeseed moisture and oil content over the storage period. As shown in Figure 1A, extending storage time significantly increased rapeseed moisture content from an initial value of 1.46% (*p* < 0.05). It is noteworthy that the packaging materials’ barrier properties critically influence the effectiveness of airtight storage. The PA/PE co-extruded composite film exhibited superior oxygen- and water-vapor-barrier performance compared to PP bags: after 52 weeks of storage, the final moisture content in the PA/PE film was 3.34%, substantially lower than that in the PP bags (5.88%). Thus, the PA/PE film offers greater advantages in maintaining safe and dry storage conditions for rapeseed. Furthermore, the oil content (49.12–48.17%) was not significantly affected by packaging type (*p* > 0.05, Figure 1B). Any slight decrease during storage may have resulted from oil leaching (oil migration from seed cells caused by cell wall swelling and structural disruption due to moisture absorption), volatilization losses of small volatile compounds, or moisture-related microbial and enzymatic metabolism—including lipase-catalyzed lipid hydrolysis producing free fatty acids, and microbial utilization of oil as a carbon source [27].

One point must be clarified here. According to the majority of recommendations, the optimum moisture content for rapeseed/canolol pressing is 5%. As demonstrated in previous work [14], the microwave pretreatment of seeds results in substantial water loss, which directly affects pressing performance. Therefore, after microwave pretreatment, seeds packaged in PA/PE co-extrusion composite film were re-moisturized up to a pressing-appropriate moisture level, consistent with that of PP-packaged rapeseeds at the same storage stage. This procedure was implemented to achieve seed moisture contents that give the highest oil yields, as well as to ensure consistency across subsequent measurements.

### 3.2. TPCs and Antioxidant Activities of Microwaved Rapeseed Seeds in PP and PA/PE Packaging During Storage

**Figure 2:**
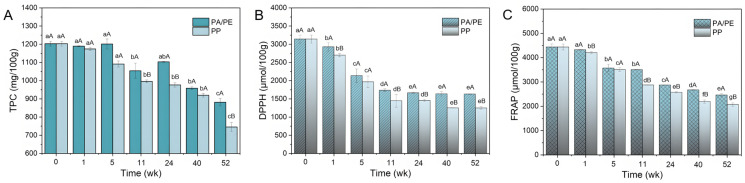
The TPCs (**A**), DPPH radical scavenging activity (**B**), and FRAP assay values (**C**) of rapeseeds during 13-month storage in PA/PE and PP packaging. Note: Different lowercase letters indicate significant (*p* < 0.05) differences over different storage periods among samples stored in the same packaging materials, while different uppercase letters indicate significant differences between different packaging materials during the same storage period (*p* < 0.05).

Among oleaginous plants, the greatest volume of phenolic compounds appears in rapeseed, with its TPC levels varying from 800 mg/100 g to even 1406 mg/100 g [6,28]; in this research, the TPC of the microwaved seed extract was 1203.05 mg/100 g. When stored at 25 °C for more than one year, the samples’ TPCs changed at different states. PA/PE-coated rapeseeds’ TPCs decreased by 0.43–26.74%, compared to a 2.40–34.66% decline in PP-coated groups (Figure 2A); it is clear that the former packaging material has a better protective effect on phenolic compounds than the latter. These results are similar to those of the previous investigation, which revealed a reduction in the phenolic traits of chickpea seeds stored at different temperatures (5, 10, 15, and 20 °C) over 540 days [29]. Rekas et al. [30] also illustrated phenolic compounds degradations in rapeseed oil over 12 months of storage. Specifically, the canolol, trans-sinapic acid, ferulic acid, sinapine, and *p*-coumaric acid contents decreased by 76.12%, 63.78%, 66.04%, 60.99%, and 68.82%, respectively.

DPPH and FRAP assays are commonly used to evaluate oilseeds’ antioxidant properties. The initial DPPH free radical scavenging ability of microwaved rapeseeds was measured at 3144.08 μmol/100 g, approximately twice the value recorded for the same sample after one year (Figure 2B). DPPH assay values remarkably decreased significantly (*p* < 0.05) across different packaging types in the early stage of storage, followed by a steady, gradual decline until week 52. A more pronounced decreasing trend was observed in FRAP total antioxidant capacity, which declined from 4438.90 μmol/100 g to 2463.51 (2074.00) μmol/100 g (Figure 2C). Moreover, both DPPH and FRAP values were consistently higher in PA/PE-coated rapeseeds compared to those with the PP coating, suggesting that PA/PE packaging better preserves antioxidant compounds, thereby offering superior oxidation resistance.

### 3.3. AV and POV Analyses of Microwaved Rapeseed Oils Packaged in PP and PA/PE During Storage

**Figure 3:**
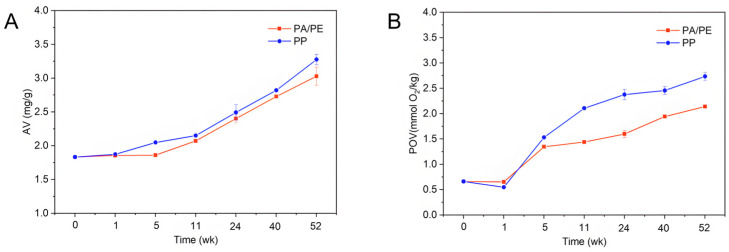
The AV (**A**) and POV (**B**) of cold-pressed rapeseed oils during 13-month storage in PA/PE and PP packaging.

Lipid oxidation poses a significant challenge to oil quality and stability across the supply chain. It compromises oil by triggering the development of detrimental components and unpalatable flavors and degrading essential fatty acids [31]. In this study, the influence of storage on homologous oil oxidation was explored by AV and POV, which represent the levels of free fatty acids (FFAs) and the primary oxidation products (hydroperoxides), respectively. As shown in Figure 3, a continuous increase in AV and POV emerged with the extension of storage time; a discernible shift occurred around week 5 following an initial phase of minimal change, with both parameters showing significant rises thereafter. On average, the oil samples’ AVs and POVs grew to 3.15 mg/g and 2.44 mmol O_2_/kg from initial values of 1.83 mg/g and 0.66 mmol O_2_/kg. The results are in agreement with the literature, in which the AV, POV, and *P*-AV of *Camellia oleifera* seed oil (COSO) progressively rose as storage duration increased, symbolizing the generation of oxidation products during accelerated storage [32]. Pristouri et al. [33] evaluated the efficacy of transparent glass, PET, and PP for oil preservation, and concluded that the free acidity and POV of extra virgin olive oil (EVOO) exhibited diverse change behaviors in different containers. In our study, PP-packaged oils exhibited significantly faster increases in both AV and POV compared to those in PA/PE (*p* < 0.05), with a range of AV increase of 2.16–78.78% for PP-packaged oils, notably higher than the 1.17–65.24% range observed for PA/PE-packaged oils. This disparity in oxidation was particularly evident by week 24 of storage. At this time point, the POV of oil stored in PP under atmospheric conditions had risen rapidly to 2.38 mmol O_2_/kg—a value that exceeded the final POV (2.14 mmol O_2_/kg) of PA/PE vacuum-packaged rapeseed oil after an entire year. The marked difference in oxidative deterioration between the two packaging systems can be directly attributed to their distinct oxygen barrier properties. The PA/PE vacuum packaging, characterized by its low oxygen permeability (proven in the previous literature [11]), effectively limited the oxygen concentration within the headspace, thereby slowing the oxidative chain reaction. Conversely, the PP packaging allowed continuous oxygen ingress under atmospheric conditions, leading to the rapid accumulation of hydroperoxides (high POV) and subsequent secondary oxidation products (e.g., aldehydes and ketones).

### 3.4. Changes in Bioactive Compounds of Microwaved Rapeseed Oils Stored in PP and PA/PE Packaging

Under long-term storage or heat treatment, oils are subjected to hydrolysis, oxidation and polymerization processes which cause the deterioration of nutritive qualities. The total tocopherol and tocopherol homologue contents of oils extracted from microwaved rapeseeds, after storage for over one year under atmospheric and vacuum conditions, are presented in Table 1. The initial pressed microwave-treated rapeseed oil had a total tocopherol content of 672.70 mg/kg, significantly higher than that of the raw oil (654.56 mg/kg) (*p* < 0.05). This finding aligns with the results reported by Cong et al. [1], who observed that microwave pretreatment increased the average total tocopherol content by 3.79% across thirty-nine rapeseed cultivars, reaching 699.50 mg/kg. At the beginning of storage (within 1 week), the oils’ total tocopherol contents showed no significant differences across packaging conditions, ranging from 665.05 to 666.51 mg/kg. The content then declined progressively until the end of the 52-week storage period. Ultimately, samples stored under atmospheric and vacuum conditions incurred losses of approximately 16.25% and 6.97%, respectively. This reduction is attributable to the self-degradation of tocopherols, which serves to protect the oil from oxidation. An evident decrease in tocopherol levels during storage under various conditions has been widely reported [9,34,35]. Chen et al. [36] confirmed that the loss of tocopherol (from 659.32 mg/kg to 509.46 mg/kg) in strong-fragrant rapeseed oils (SFROs) after 6-month oxidation was potentially due to their consumption to counteract oxidative stress excited by chlorophyll at wavelength of 680 nm (red light). Another study verified vacuum packaging as effective in maintaining quality during the extended storage of dry roasted peanuts based on oxidative changes and retention of vitamin E [9]. Furthermore, *α*-tocopherol degraded more rapidly than *γ*-tocopherol under both packaging strategies, due to the lower oxidative stability of *α*-tocopherol and its faster reaction with peroxy radicals [15,37]. Specifically, the degradation rates of *α*-tocopherol and *γ*-tocopherol in the PP-coated samples (18.07% and 3.90%) were 2.14 and 2.04 times higher, respectively, than those in the PA/PE-coated samples (8.46% and 1.91%).

Phytosterol is the other typical micronutrient found in oil besides tocopherol, and its biological properties—particularly the capacity to reduce blood cholesterol levels—have attracted considerable interest on the part of the pharmaceutical and food industries. Table 1 illustrates the individual phytosterols and total sterol levels in rapeseed oils throughout our experiment. In fresh microwaved rapeseed oil, the total phytosterol content was 6674.27 g/kg, comprising 46.27% sitosterol, 41.08% campesterol and 16.64% brassicasterol. During storage, phytosterol levels fluctuated, distinct from the typically monotonic decline observed in tocopherols. This fluctuation can be attributed to several analytical and physicochemical factors. Phytosterols might undergo physical entrapment within complex matrices or interact with oxidized triglycerides, phospholipids, or proteins, leading to inconsistent recovery rates during sample pretreatment steps such as saponification and extraction [38]. Concurrently, phytosterols could isomerize into compounds (stanenols), which were not fully separated or accurately quantified by conventional analytical methods [39]. These combined effects resulted in variable extraction efficiencies across measurements, ultimately manifesting as apparent fluctuations in phytosterol content in the analytical data.

Canolol is assumed to be generated when sinapic acid is heat decarboxylated on exposure to microwave or heat treatment. It is well-established, as reported by Spielmeyer et al. [40], that microwave pretreatment (560 W, 7.5 min, 160 °C) can increase canolol content by up to 120-fold; in alignment with this, we observed a 26-fold increase in canolol levels after seed microwaving. Given this significant enhancement, we subsequently focused on evaluating the influence of packaging materials and storage time on canolol stability (Table 1). The initial canolol content in the microwave-pretreated rapeseed oil was 1411.18 mg/kg, and its degradation exhibited significant packaging-dependent trends over the 52 weeks. PA/PE vacuum packaging (low oxygen permeability) reduced canolol loss to 22.41% at the end of storage, compared to 76.34% in PP atmospheric packaging. Significant divergence appeared as early as week 5 (6.74% vs. 19.33% loss), directly demonstrating that oxygen ingress is the key trigger for canolol degradation and subsequent tocopherol depletion. A similar relationship between the residual canolol content and its initial concentration was reported by Rękas et al. [30]. In the long-term storage test of rapeseed oils prepared from microwave-pretreated seeds (6, 10 min, 800 W), the respective canolol concentrations decreased to 31.1 and 996.7 mg/kg from 286.96 and 1692.15 mg/kg, respectively. Pearson correlation analysis confirmed the critical role of canolol in inhibiting oil oxidation, as canolol content was strongly negatively correlated with AV (*r* = −0.984, *p* < 0.01) and POV (*r* = −0.959, *p* < 0.01). Furthermore, a significant positive correlation was observed between canolol and total tocopherol content (*r* = 0.966, *p* < 0.01), confirming the synergistic antioxidant effect between the two. Canolol has a higher phenolic hydroxyl group reactivity than tocopherols [41], enabling it to preferentially scavenge lipid peroxyl radicals and reduce tocopherol consumption. In PP-packaged oil (high oxygen permeability), drastic canolol degradation leads to the loss of this protective effect, and tocopherols are forced to act as the primary antioxidant, resulting in accelerated degradation. In contrast, PA/PE vacuum packaging (low oxygen permeability) preserves canolol activity, maintaining the synergistic antioxidant system and effectively reduces tocopherol loss. This finding clarifies the underlying mechanism of the synergistic effect between canolol and tocopherols in rapeseed oil, and further emphasizes the core status of canolol in rapeseed oil’s antioxidant system.

### 3.5. HPLC-Q-TOF/MS/MS Analysis of the Antioxidant Products of Canolol

The antioxidant behaviour of canolol was evaluated in a lipid medium containing EtL (3.0 g) as the oxidizable substrate and AIBN (0.6 g) as the radical initiator, dissolved in CH_3_CN solution (3 mL) and incubated at 40 °C. In addition to the canolol signal (peak 1), one additional peak (peak 2) appeared in the total ion chromatogram (TIC) after 6 h, which was absent at 0 h (Figure S1). The structural elucidation of the antioxidant products of canolol was carried out by comparing their accurate mass and mass fragmentation data. The most plausible structures for two antioxidant products can be arrived at with the help of the HPLC-Q-TOF/MS/MS data listed in Table 2; the characterization of each is discussed below individually.

### 3.6. Proposed Antioxidation Mechanism of Canolol in EtL

After incubating canolol with AIBN in acetonitrile in the presence of EtL under air at 40 °C for 6 h, LC-Triple-QTOF-MS/MS analysis in information-dependent acquisition (IDA) mode with negative ESI revealed a prominent ion at *m*/*z* 375.1437. The elemental composition C_20_H_24_O_7_ to the ion was assigned based on its accurate mass (calcd [M-H]^−^ 375.1449; Δ = −3.3 ppm), suggesting the formation of a canolol-derived dimeric oxidation product.

The MS/MS spectrum showed a predominant fragment ion at *m*/*z* 357.1314, corresponding to a neutral loss of 18 Da, which is consistent with the elimination of water ([M-H-H_2_O]^−^) and indicates the presence of a labile aliphatic hydroxyl group, potentially originating from a hydroxyethyl moiety formed via vinyl group functionalization. Subsequent fragment ions at *m*/*z* 342.1079 and 327.0833, representing successive losses of approximately 15 Da, are characteristic of the stepwise elimination of methyl radicals (•CH_3_) from methoxy substituents under collision-induced dissociation, a typical fragmentation behavior of methoxylated phenolic compounds such as those bearing syringyl units. Further structural evidence was provided by diagnostic low-mass fragment ions at *m*/*z* 181.0500 (C_9_H_9_O_4_^−^), 166.0263 (C_8_H_6_O_4_^−^), and 151.0025 (C_7_H_3_O_4_^−^), which correspond to syringyl-type anions commonly observed in the fragmentation of oxidized dimethoxy-hydroxy aromatic compounds.

**Figure 4:**
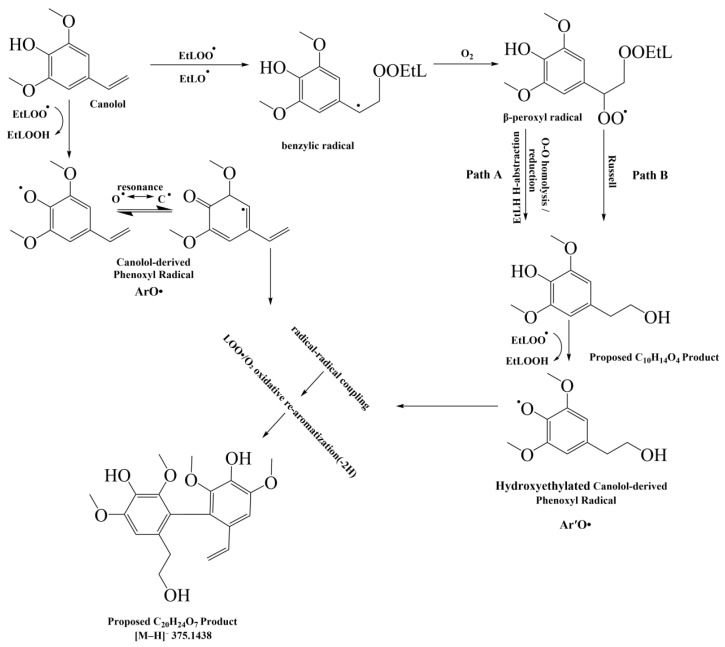
A scheme of the proposed canolol antioxidation mechanism in EtL.

Mechanistically, the AIBN/EtL/air system at 40 °C the AIBN/EtL/air system at 40 °C establishes a lipid autoxidation environment where lipid-derived peroxyl radicals and hydroperoxides are continuously generated [42]. Under these conditions, canolol functions primarily as a chain-breaking antioxidant via hydrogen atom transfer (HAT) from its phenolic hydroxyl group to lipid-derived peroxyl radicals (EtLOO• + ArOH → EtLOOH + ArO•), yielding a resonance-stabilized canolol-derived phenoxyl radical (ArO•) that is susceptible to oxidative coupling [43]. In a parallel pathway, lipid-derived radical species may add to the electron-rich vinyl substituent of canolol, generating a carbon-centered (benzylic) radical that retains the parent C_10_ skeleton. Subsequent trapping of this carbon-centered radical by molecular oxygen affords the corresponding peroxyl radical (ROO•), with the unpaired electron located on the terminal oxygen atom [44]. This peroxyl intermediate may undergo further transformation via two possible routes (Figure 4). Path A: HAT from a lipid substrate (EtL) yields a hydroperoxide, which upon O-O bond homolysis and subsequent HAT may furnish a hydroxyethylated canolol derivative (Ar′OH, C_10_H_14_O_4_). Path B: Self- or cross-termination of two peroxyl radicals via a Russell-type mechanism, proceeding through a tetroxide intermediate, may produce one molecule each of alcohol, carbonyl compound (aldehyde or ketone), and singlet oxygen (^1^O_2_) [45,46]. The observed C_20_H_24_O_7_ species is therefore proposed to form via oxidative cross-coupling between canolol-derived radical intermediates, specifically the phenoxyl radical (ArO•) and a hydroxyethylated canolol-derived phenoxyl radical (Ar′O•). Based on accurate mass measurements and the observed dehydration and methoxy-loss fragments, the ion at *m*/*z* 375.1437 was tentatively assigned as a canolol-derived dimeric oxidation product. Its fragmentation pattern is consistent with an oxygenated dimer containing syringyl-type aromatic units and aliphatic hydroxyl functionality, possibly arising from oxidative cross-coupling between a vinyl-canolol-derived unit and a hydroxyethylated canolol-derived unit.

## 4. Conclusions

This study proposes a plausible molecular mechanism for canolol’s antioxidant action in a lipid autoxidation model system and demonstrates its practical preservation through optimized packaging. The central finding is the detection and tentative characterization—via HPLC-Q-TOF/MS/MS—of a canolol-derived dimeric oxidation product with the elemental composition C_20_H_24_O_7_ (*m*/*z* 375.1437), providing direct mass spectrometric evidence for the formation of canolol-derived oxidation products in a lipid autoxidation system. The MS/MS fragmentation pattern (neutral H_2_O loss, sequential •CH_3_ eliminations, syringyl-type diagnostic ions) is consistent with a mechanism involving hydrogen atom transfer followed by oxidative cross-coupling between a canolol-derived phenoxyl radical (ArO•) and a hydroxyethylated canolol-derived radical intermediate (Ar′O•), supporting canolol’s role as a chain-breaking antioxidant. From an applied perspective, we demonstrate that microwave pretreatment combined with PA/PE vacuum packaging maximizes canolol retention (22.41% loss vs. 76.34% in PP) and preserves its antioxidant functionality over 52 weeks. The strong correlations between canolol content and both oxidative indices (*r* = −0.914 to −0.984 for AV/POV) and tocopherol stability (*r* = 0.878–0.966) validate that canolol preservation directly translates to enhanced oil stability. By bridging molecular mechanism identification with long-term storage optimization, this work provides a scientific foundation for producing cold-pressed rapeseed oil with superior oxidative stability and nutritional quality.

## Figures and Tables

**Table 1:** The contents of tocopherol, phytosterol and canolol in cold-pressed rapeseed oils during 13-month storage in PA/PE and PP packaging.

Storage Time (wk)	*α*-Tocopherol (mg/kg)		*γ*-Tocopherol (mg/kg)		Total Tocopherol (mg/kg)		Brassicasterol (mg/kg)		Campesterol (mg/kg)		*β*-Sitosterol (mg/kg)		Total Phytosterol (mg/kg)		Canolol (mg/kg)	
	PA/PE	PP	PA/PE	PP	PA/PE	PP	PA/PE	PP	PA/PE	PP	PA/PE	PP	PA/PE	PP	PA/PE	PP
0	238.57 ± 0.42 ^aA^	238.57 ± 0.42 ^aA^	434.15 ± 3.15 ^aA^	434.15 ± 3.15 ^aA^	672.71 ± 3.57 ^aA^	672.71 ± 3.57 ^aA^	843.90 ± 3.23 ^cA^	843.90 ± 3.23 ^dA^	2742.07 ± 1.74 ^cdA^	2742.07 ± 1.74 ^cA^	3088.31 ± 2.61 ^bA^	3088.31 ± 2.61 ^dA^	6674.27 ± 6.88 ^cA^	6674.27 ± 6.88 ^cA^	1411.18 ± 4.05 ^aA^	1411.18 ± 4.05 ^aA^
1	235.56 ± 1.57 ^aA^	235.90 ± 0.64 ^aA^	429.49 ± 1.83 ^aA^	430.61 ± 1.92 ^abA^	665.05 ± 3.32 ^aA^	666.51 ± 1.28 ^aA^	860.34 ± 9.75 ^cA^	855.07 ± 9.93 ^dA^	2761.90 ± 30.65 ^bcA^	2774.72 ± 35.43 ^bcA^	3098.88 ± 28.20 ^bA^	3117.76 ± 38.91 ^cdA^	6721.12 ± 61.06 ^bcA^	6747.55 ± 83.65 ^cA^	1409.85 ± 6.26 ^aA^	1380.35 ± 12.48 ^bB^
5	236.37 ± 1.71 ^aA^	227.99 ± 3.94 ^bB^	428.58 ± 6.38 ^aA^	424.69 ± 5.32 ^bcB^	664.96 ± 8.02 ^aA^	652.68 ± 6.54 ^bB^	894.17 ± 10.92 ^aA^	907.09 ± 12.95 ^bcA^	2820.98 ± 21.98 ^abA^	2838.60 ± 40.40 ^bA^	3199.81 ± 8.21 ^aA^	3208.49 ± 46.36 ^bcA^	6914.96 ± 32.77 ^abA^	6954.18 ± 99.62 ^bA^	1316.06 ± 6.38 ^bA^	1138.33 ± 4.75 ^cB^
11	232.83 ± 5.97 ^aA^	218.26 ± 4.37 ^cB^	416.87 ± 2.16 ^bA^	405.55 ± 1.77 ^dB^	649.70 ± 6.89 ^bA^	623.80 ± 3.13 ^cB^	853.78 ± 22.91 ^cB^	922.64 ± 18.46 ^abA^	2735.22 ± 47.58 ^cdB^	2935.08 ± 54.95 ^aA^	3135.15 ± 54.55 ^abB^	3345.02 ± 60.21 ^aA^	6724.15 ± 71.82 ^cB^	7202.74 ± 132.88 ^aA^	1272.26 ± 5.24 ^cA^	1116.43 ± 12.36 ^dB^
24	213.20 ± 3.93 ^bA^	195.96 ± 6.92 ^dB^	418.94 ± 7.92 ^bA^	404.83 ± 5.91 ^dB^	632.15 ± 11.61 ^cA^	600.79 ± 1.01 ^dB^	886.31 ± 10.22 ^abA^	898.99 ± 8.40 ^cA^	2737.79 ± 12.58 ^cdA^	2772.23 ± 44.67 ^bcA^	3121.42 ± 28.73 ^bA^	3047.45 ± 8.53 ^deB^	6745.52 ± 45.88 ^bcA^	6718.67 ± 60.16 ^cA^	1195.70 ± 11.21 ^dA^	924.34 ± 7.21 ^eB^
40	194.11 ± 1.35 ^cA^	149.72 ± 6.77 ^eB^	429.50 ± 1.52 ^aA^	419.05 ± 0.84 ^cB^	623.61 ± 2.68 ^cA^	568.77 ± 6.40 ^eB^	889.58 ± 8.28 ^aB^	936.53 ± 5.91 ^aA^	2857.30 ± 12.02 ^aB^	2923.44 ± 18.61 ^aA^	3090.23 ± 24.31 ^bB^	3277.72 ± 10.74 ^abA^	6837.10 ± 44.56 ^abB^	7137.69 ± 31.39 ^aA^	1215.88 ± 8.72 ^eA^	512.11 ± 3.80 ^fB^
52	193.99 ± 4.12 ^cA^	144.86 ± 3.40 ^eB^	431.81 ± 7.90 ^aA^	418.54 ± 2.43 ^cB^	625.81 ± 11.66 ^cA^	563.40 ± 3.49 ^eB^	864.83 ± 12.17 ^bcA^	848.61 ± 6.40 ^dB^	2684.27 ± 81.58 ^dA^	2667.58 ± 51.73 ^dA^	3108.41 ± 70.21 ^bA^	2983.58 ± 111.41 ^eA^	6657.52 ± 151.68 ^cA^	6499.77 ± 160.87 ^dA^	1094.96 ± 12.56 ^fA^	333.92 ± 8.76 ^gB^

Note: Different lowercase letters indicate significant (*p* < 0.05) differences over different storage periods among samples stored in the same packaging materials, while uppercase letters indicate significant differences between different packaging materials during the same storage period (*p* < 0.05).

**Table 2:** The radical reaction products of canolol as determined by HPLC-Q-TOF-MS/MS.

RT[min]	Accurate Mass	Exact Mass of Most Probable Structure	Error in ppm	RDB	*m*/*z* of Main Fragments by ESI-MS^2^	Molecular Formula
14.05	179.0706	179.0714	−4.3	5	149.0245, 121.0301, 164.0473, 93.0360	C_10_H_12_O_3_
17.09	375.1437	375.1449	−3.3	9	181.0500, 166.0263, 342.1079, 327.0833, 357.1314, 151.0025, 178.0629, 192.0789	C_20_H_24_O_7_

## Data Availability

The original contributions presented in this study are included in the article/Supplementary Materials. Further inquiries can be directed to the corresponding author.

## Supplementary Materials

The following supporting information can be downloaded at: https://www.mdpi.com/article/10.3390/foods15101797/s1, Figure S1. Total ion chromatograms of the ethyl linoleate/AIBN/CH_3_CN system after 6 h of reaction: (A) without canolol; (B) with canolol.

## Abbreviations

The following abbreviations are used in this manuscript:

PUFA	Polyunsaturated fatty acids
Canolol	2,6-Dimethoxy-4-vinylphenol
*P*-AV	*P*-anisidine value
POV	Peroxide value
PET	Polyethylene terephthalate
AL	Aluminum
PA	Polyamide
PE	Polyethylene
PA/PE	Polyamide/polyethylene
PP	Polypropylene
AV	Acid value
AIBN	2,2’-Azobis(isobutyronitrile)
EtL	Ethyl linoleate
HPLC-Q-TOF/MS/MS	High-performance liquid chromatography-quadrupole time-of-flight tandem mass spectrometry
BSTFA + TMCS	N,O-Bis(trimethylsilyl)trifluoroacetamide with trimethylchlorosilane
FC	Folin–Ciocalteu
DPPH	2,2-dipheny1-1-picrylhydrazyl
TPTZ	2,4,6-tris(2-pyridyl)-s-triazine
Trolox	6-hydroxy-2,5,7,8-tetramethylchroman-2-carboxylic acid
TPC	Total phenolic content
SAE	Sinapic acid equivalent
TE	Trolox equivalent
FRAP	Ferric reducing antioxidant power
HPLC	High-performance liquid chromatography
GC	Gas chromatography
UPLC	Ultra-performance liquid chromatography
PDA	Photodiode array
ESI	Electrospray ionization
FFA	Free fatty acid
COSO	*Camellia oleifera* seed oil
EVOO	Extra virgin olive oil
SFRO	Strong-fragrant rapeseed oils
TIC IDA	Total ion chromatogram Information-dependent acquisition
HAT	Hydrogen atom transfer

## References

[1] Cong Y., Zheng M., Huang F., Liu C., Zheng C. (2020). Sinapic acid derivatives in microwave-pretreated rapeseeds and minor components in oils. J. Food Compos. Anal..

[2] Quiles J.L., Ramírez-Tortosa M.C., Gómez J.A., Huertas J.R., Mataix J. (2002). Role of vitamin E and phenolic compounds in the antioxidant capacity, measured by ESR, of virgin olive, olive and sunflower oils after frying. Food Chem..

[3] Thiyam U., Stöckmann H., Zum Felde T., Schwarz K. (2006). Antioxidative effect of the main sinapic acid derivatives from rapeseed and mustard oil by-products. Eur. J. Lipid Sci. Technol..

[4] Koski A., Pekkarinen S., Hopia A., Wähälä K., Heinonen M. (2003). Processing of rapeseed oil: Effects on sinapic acid derivative content and oxidative stability. Eur. Food Res. Technol..

[5] Aachary A.A., Chen Y., Eskin N.A.M., Thiyam-Hollander U. (2014). Crude canolol and canola distillate extracts improve the stability of refined canola oil during deep-fat frying. Eur. J. Lipid Sci. Technol..

[6] Mayengbam S., Aachary A., Thiyam-Hollander U. (2014). Endogenous Phenolics in Hulls and Cotyledons of Mustard and Canola: A Comparative Study on Its Sinapates and Antioxidant Capacity. Antioxidants.

[7] Shrestha K., De Meulenaer B. (2014). Effect of seed roasting on canolol, tocopherol, and phospholipid contents, Maillard type reactions, and oxidative stability of mustard and rapeseed oils. J. Agric. Food Chem..

[8] Zheng C., Yang M., Zhou Q., Liu C.S., Huang F.H. (2014). Changes in the content of canolol and total phenolics, oxidative stability of rapeseed oil during accelerated storage. Eur. J. Lipid Sci. Technol..

[9] Chun J., Lee J., Eitenmiller R.R. (2006). Vitamin E and Oxidative Stability During Storage of Raw and Dry Roasted Peanuts Packaged under Air and Vacuum. J. Food Sci..

[10] Severini C., Pill1 T.D., Baiano A. (2003). Autoxidation of packed roasted almonds as affected by two packaging films. J. Food Process. Preserv..

[11] Fu X., Xing S., Xiong H., Min H., Zhu X., He J., Feng J., Mu H. (2018). Effects of packaging materials on storage quality of peanut kernels. PLoS ONE.

[12] Azadmard-Damirchi S., Habibi-Nodeh F., Hesari J., Nemati M., Achachlouei B.F. (2010). Effect of pretreatment with microwaves on oxidative stability and nutraceuticals content of oil from rapeseed. Food Chem..

[13] Yang M., Huang F., Liu C., Zheng C., Zhou Q., Wang H. (2012). Influence of Microwave Treatment of Rapeseed on Minor Components Content and Oxidative Stability of Oil. Food Bioprocess Technol..

[14] Wroniak M., Rękas A., Siger A., Janowicz M. (2016). Microwave pretreatment effects on the changes in seeds microstructure, chemical composition and oxidative stability of rapeseed oil. LWT-Food Sci. Technol..

[15] Abramovič H., Butinar B., Nikolič V. (2007). Changes occurring in phenolic content, tocopherol composition and oxidative stability of *Camelina sativa* oil during storage. Food Chem..

[16] Pan F., Wang X., Wen B., Wang C., Xu Y., Dang W., Zhang M. (2020). Development of walnut oil and almond oil blends for improvements in nutritional and oxidative stability. Grasas Aceites.

[17] Gawrysiak-Witulska M., Rudzinska M., Wawrzyniak J., Siger A. (2012). The Effect of Temperature and Moisture Content of Stored Rapeseed on the Phytosterol Degradation Rate. J. Am. Oil Chem. Soc..

[18] Lang C., Huang Y., Lin K., Chen W., Chen W., Zhong Q., Pei J., Lv Y., He R., Zhang M. (2025). Exploring the optimization of microwave-treated rapeseed oil extraction based on response surface and lipidomics and its effects on quality characteristics, chemical composition, nutritional properties, and antioxidant capacity during storage. J. Food Compos. Anal..

[19] (2016). Determination of Moisture in Foods.

[20] (2016). Determination of Fat in Foods.

[21] (2017). Acid Value.

[22] (2016). Determination of Peroxide Value in Foods.

[23] Wang W., Yang B., Li W., Zhou Q., Liu C., Zheng C. (2021). Effects of steam explosion pretreatment on the bioactive components and characteristics of rapeseed and rapeseed products. LWT-Food Sci. Technol..

[24] Ma X., Zheng C., Zhou Q., Huang C., Wang W., Huang Y., Liu C. (2024). Comparison evaluation pretreatments on the quality characteristics, oxidative stability, and volatile flavor of walnut oil. Food Chem..

[25] Wang W., Zheng C., Yang B., Li W., Huang F., Liu C. (2025). Effect of radio frequency pretreatment on the component of rapeseed and its product: Comparative study with microwave pretreatment under different oil extraction methods. Food Chem..

[26] Huang Y., Liu C., Ge Z., Huang F., Tang H., Zhou Q., Liu R., Huang J., Zheng C. (2023). Influence of different thermal treatment methods on the processing qualities of sesame seeds and cold-pressed oil. Food Chem..

[27] Yang Y., Chen J., Chen L. (2025). A comprehensive insight into peanut storage: Patterns of quality changes, pathways of quality deterioration, and storage strategies. Food Chem. X.

[28] Cong Y., Cheong L.-Z., Huang F., Zheng C., Wan C., Zheng M. (2019). Effects of microwave irradiation on the distribution of sinapic acid and its derivatives in rapeseed and the antioxidant evaluation. LWT-Food Sci. Technol..

[29] Yeken M.Z., Soydemir H.E., Kibar H., Çiftçi V. (2023). Long-term storage affects on the phenolic, mineral, color and cooking traits of chickpea seed. J. Stored Prod. Res..

[30] Rękas A., Wroniak M. (2018). Oxidation kinetics of rapeseed oil pressed from microwave pre-treated seeds during long-term storage. J. Food Process. Preserv..

[31] Xu Y.-J., Jiang F., Song J., Yang X., Shu N., Yuan L., Tan C.P., Liu Y. (2020). Understanding of the Role of Pretreatment Methods on Rapeseed Oil from the Perspective of Phenolic Compounds. J. Agric. Food Chem..

[32] Zhao X., Zhang H., Wang S., Zhang X., Zhong W., Wang X., Gao P. (2026). Optimizing oxidative stability of Camellia oleifera seed oil: Multimodal analysis of antioxidant efficacy, tocopherol loss, benzo[a]pyrene suppression, and kinetic mechanisms. Food Chem..

[33] Pristouri G., Badeka A., Kontominas M.G. (2010). Effect of packaging material headspace, oxygen and light transmission, temperature and storage time on quality characteristics of extra virgin olive oil. Food Control.

[34] Yao F., Dull G., Eitenmiller R. (1992). Tocopherol Quantification by HPLC in Pecans and Relationship to Kernel Quality during Storage. J. Food Sci..

[35] Erickson M.C., Santerre C.R., Malingre M.E. (1994). Oxidative Stability in Raw and Roasted Pecans: Chemical, Physical and Sensory Measurements. J. Food Sci..

[36] Chen C., Ye P.-P., Cui F.-J., Tan M., Zhang H.-B., Zhou T.-L., Shi J.-C., Shu X.-Q., Chen Z.-W. (2024). Overall quality changes and deterioration mechanism of fragrant rapeseed oils during 6-Month storage. Food Chem..

[37] Gawrysiak-Witulska M., Siger A., Wawrzyniak J., Nogala-Kalucka M. (2011). Changes in tocochromanol content in seeds of brassica napus l. During adverse conditions of storage. J. Am. Oil Chem. Soc..

[38] Evtyugin D.D., Evtuguin D.V., Casal S., Domingues M.R. (2023). Advances and Challenges in Plant Sterol Research: Fundamentals, Analysis, Applications and Production. Molecules.

[39] Schröder M., Vetter W. (2012). Investigation of unsaponifiable matter of plant oils and isolation of eight phytosterols by means of high-speed counter-current chromatography. J. Chromatogr. A.

[40] Spielmeyer A., Wagner A., Jahreis G. (2009). Influence of thermal treatment of rapeseed on the canolol content. Food Chem..

[41] Siger A., Michalak M. (2015). The long-term storage of cold-pressed oil from roasted rapeseed: Effects on antioxidant activity and levels of canolol and tocopherols. Eur. J. Lipid Sci. Technol..

[42] Litwinienko G., Dąbrowska M. (2001). Thermogravimetric investigation of antioxidant activity of selected compounds in lipid oxidation. J. Therm. Anal. Calorim..

[43] Wakamatsu D., Morimuraa T., Kida K., Nakai C., Maeda H. (2005). Isolation, identification, and structure of a potent alkyl-peroxyl radical scavenger in crude canola oil, canolol. Biosci. Biotechnol. Biochem..

[44] Do Q., Lee D.D., Dinh A.N., Seguin R.P., Zhang R., Xu L. (2021). Development and application of a peroxyl radical clock approach for measuring both hydrogen-atom transfer and peroxyl radical addition rate constants. J. Org. Chem..

[45] Russell G.A. (1957). Deuterium-isotope effects in the autoxidation of aralkyl hydrocarbons. II. The interaction of peroxy radicals. J. Am. Chem. Soc..

[46] Lindsay D., Howard J.A., Horswill E.C., Iton L., Ingold K.U., Cobbley T., Ll A. (1973). The bimolecular self-reactions of secondary peroxy radicals. Product studies. Can. J. Chem..

