# Supplementary material for: Unveiling the Antioxidant Mechanism of Canolol: Packaging Impacts the Long-Term Stability of Microwave-Pretreated Rapeseed Oil
Source: Foods. 2026 May 19;15(10):1797. doi: 10.3390/foods15101797 (PMC13205409; doi:10.3390/foods15101797)

Figure S1. Total ion chromatograms of the ethyl linoleate/AIBN/CH_3CN system after 6 h of reaction: (A) without canolol; (B) with canolol.

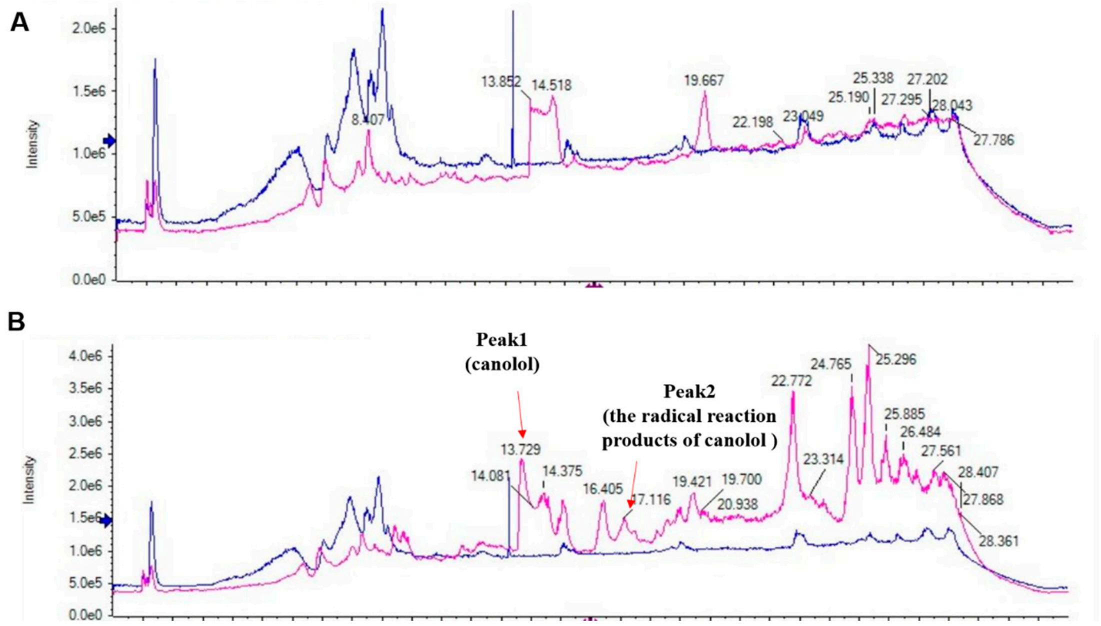

Supplement: Supplementary file 1 [file foods-15-01797-s001.zip › foods-4214587-supplementary.pdf]
